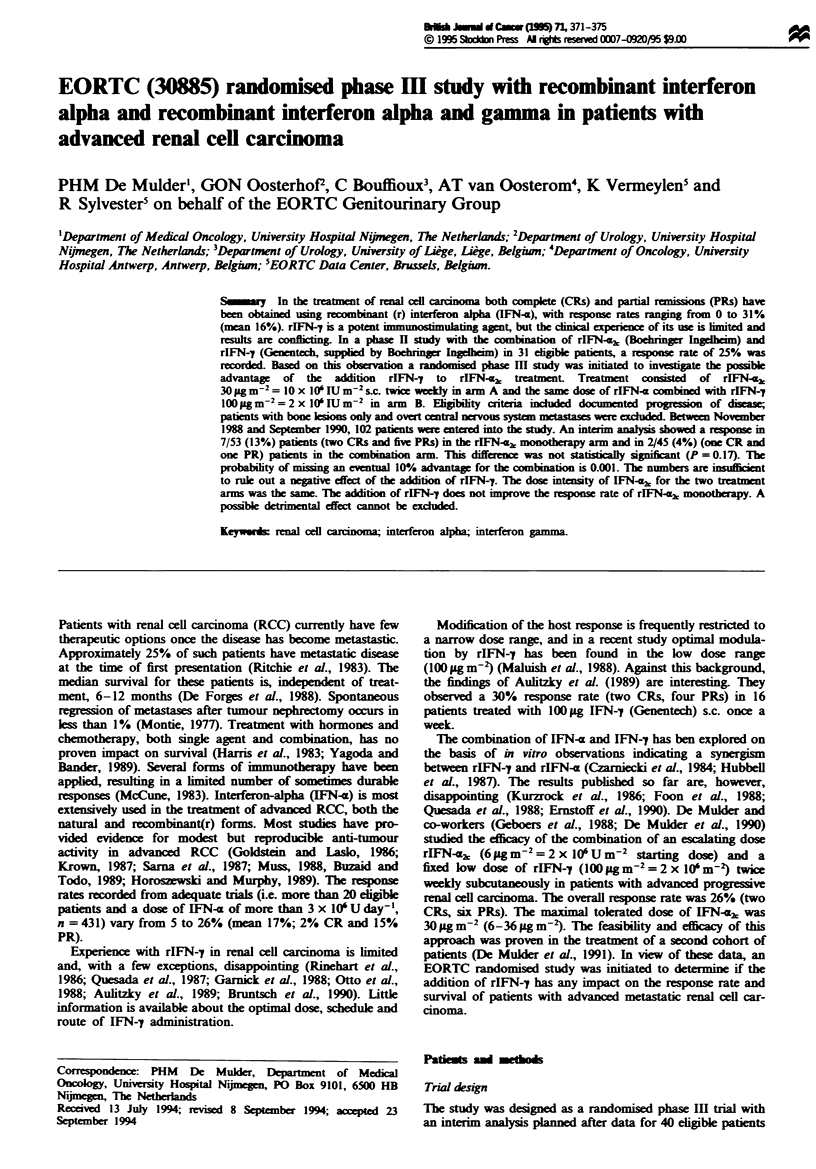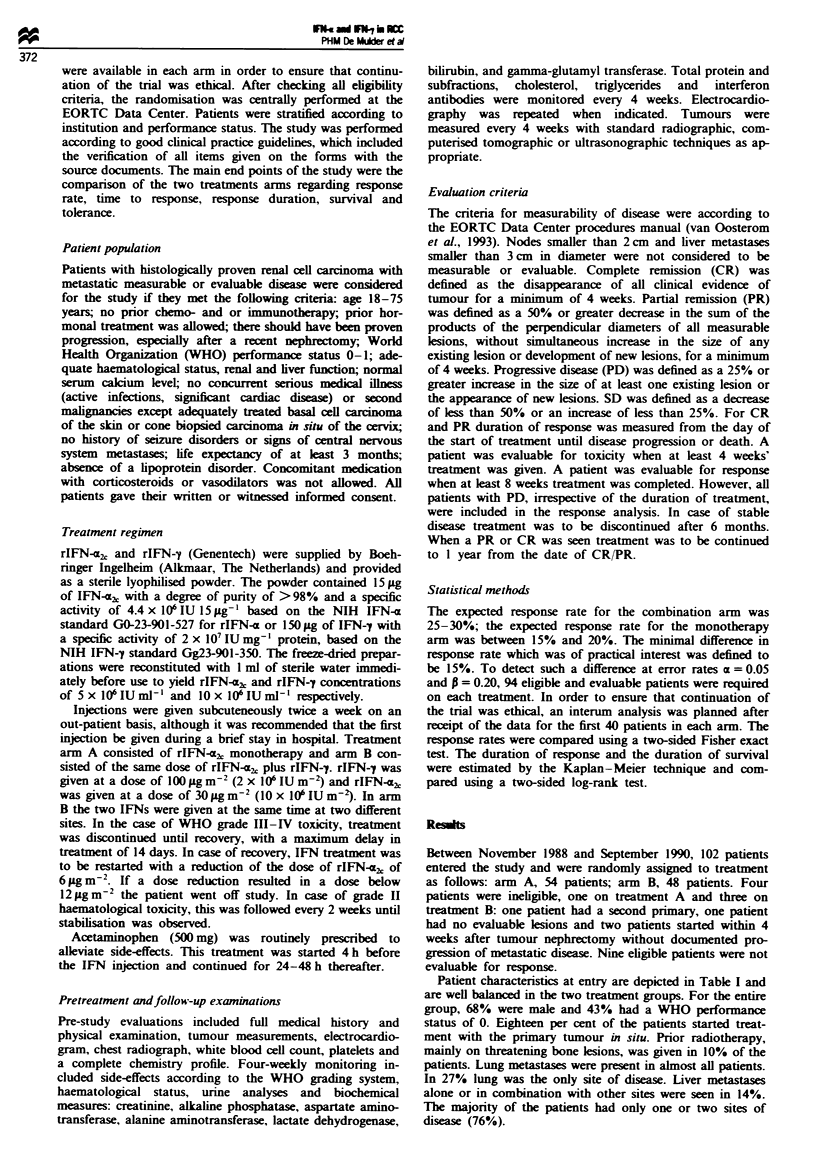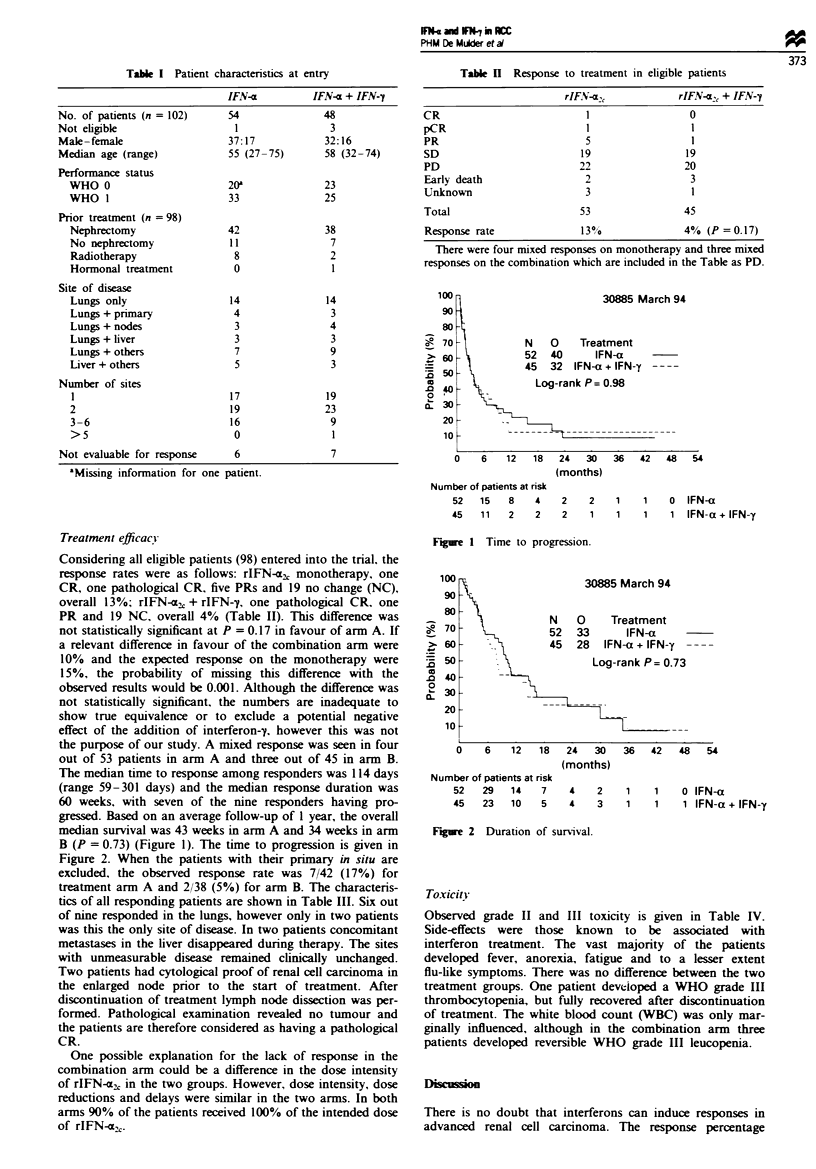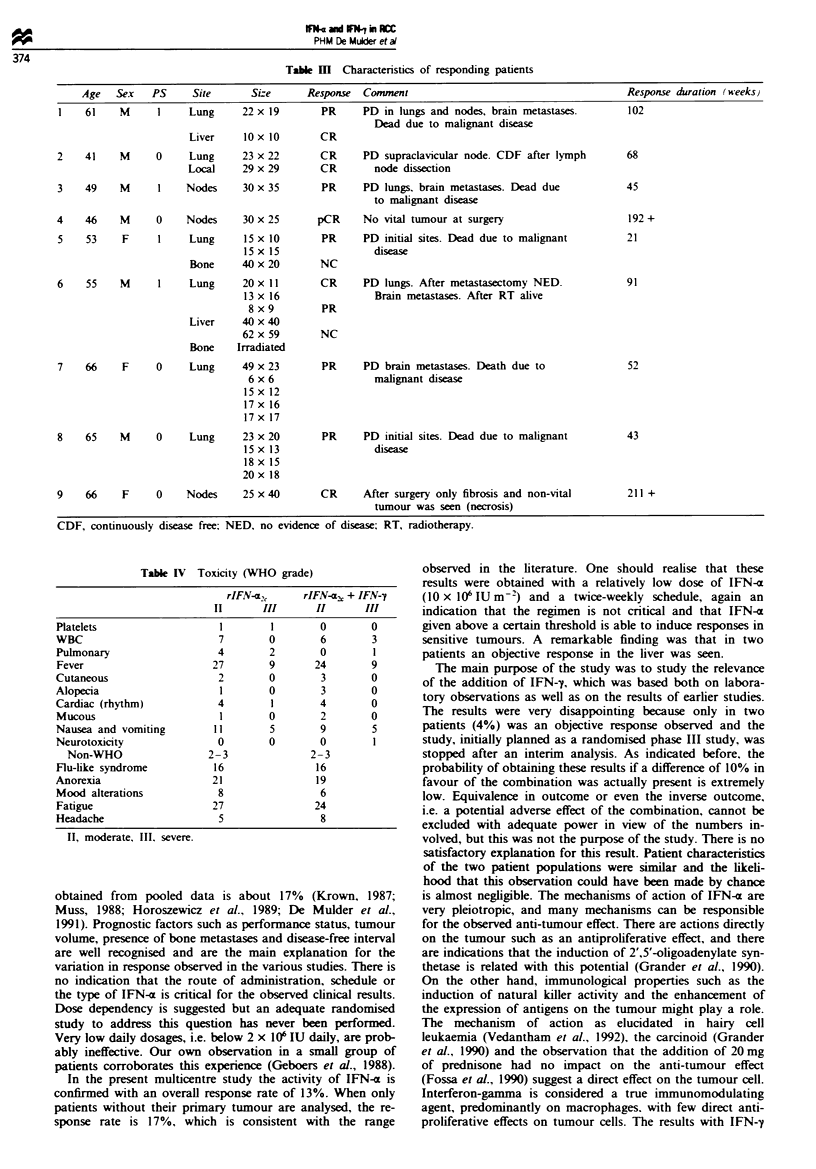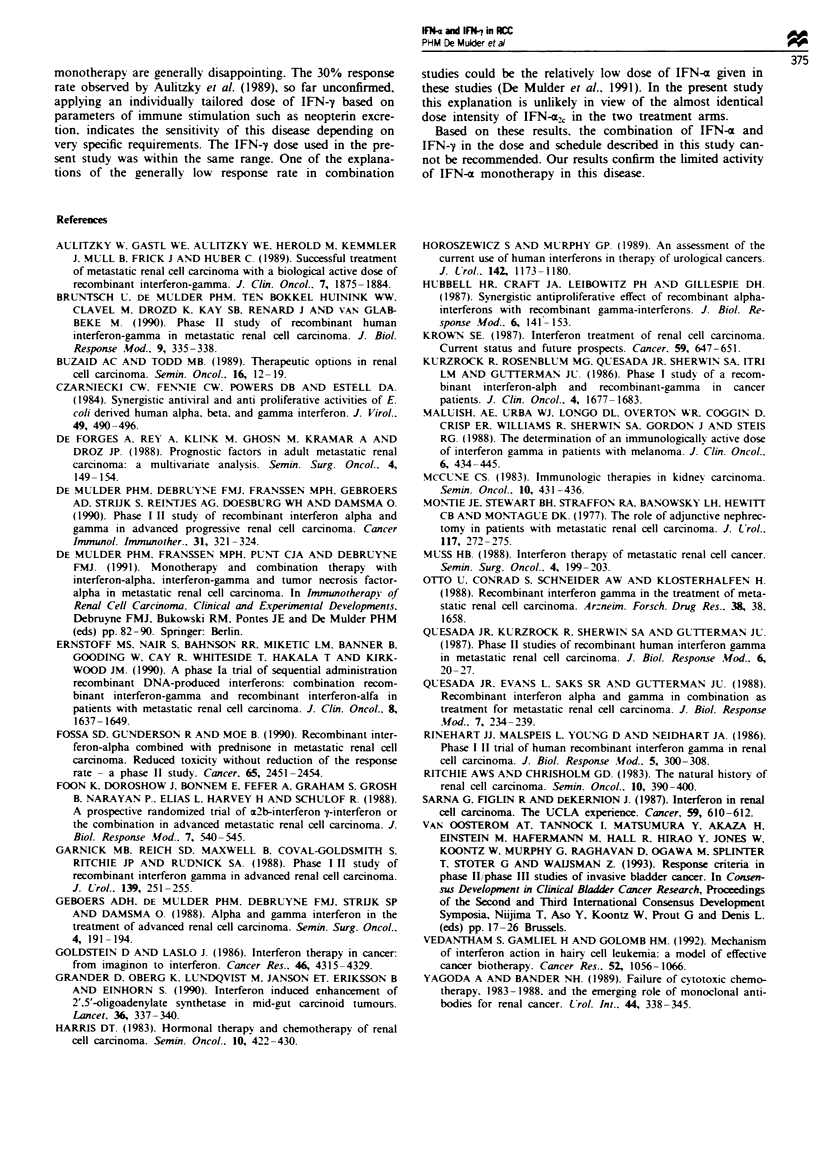# EORTC (30885) randomised phase III study with recombinant interferon alpha and recombinant interferon alpha and gamma in patients with advanced renal cell carcinoma. The EORTC Genitourinary Group.

**DOI:** 10.1038/bjc.1995.75

**Published:** 1995-02

**Authors:** P. H. De Mulder, G. Oosterhof, C. Bouffioux, A. T. van Oosterom, K. Vermeylen, R. Sylvester

**Affiliations:** Department of Medical Oncology, University Hospital Nijmegen, The Netherlands.

## Abstract

In the treatment of renal cell carcinoma both complete (CRs) and partial remissions (PRs) have been obtained using recombinant (r) interferon alpha (IFN-alpha), with response rates ranging from 0 to 31% (mean 16%). rIFN-gamma is a potent immunostimulating agent, but the clinical experience of its use is limited and results are conflicting. In a phase II study with the combination of rIFN-alpha 2c (Boehringer Ingelheim) and rIFN-gamma (Genentech, supplied by Boehringer Ingelheim) in 31 eligible patients, a response rate of 25% was recorded. Based on this observation a randomised phase III study was initiated to investigate the possible advantage of the addition rIFN-gamma to rIFN-alpha 2c treatment. Treatment consisted of rIFN-alpha 2c 30 micrograms m-2 = 10 x 10(6) IU m-2 s.c. twice weekly in arm A and the same dose of rIFN-alpha combined with rIFN-gamma 100 micrograms m-2 = 2 x 10(6) IU m-2 in arm B. Eligibility criteria included documented progression of disease; patients with bone lesions only and overt central nervous system metastases were excluded. Between November 1988 and September 1990, 102 patients were entered into the study. An interim analysis showed a response in 7/53 (13%) patients (two CRs and five PRs) in the rIFN-alpha 2c monotherapy arm and in 2/45 (4%) (one CR and one PR) patients in the combination arm. This difference was not statistically significant (P = 0.17). The probability of missing an eventual 10% advantage for the combination is 0.001. The numbers are insufficient to rule out a negative effect of the addition of rIFN-gamma. The dose intensity of IFN-alpha 2c for the two treatment arms was the same. The addition of rIFN-gamma does not improve the response rate of rIFN-alpha 2c monotherapy. A possible detrimental effect cannot be excluded.


© 1995 Stockton Press All rights reserved 0007-0920/95 $9.00

### EORTC (30885) randomised phase III study with recombinant interferon alpha and recombinant interferon alpha and gamma in patients with advanced renal cell carcinoma

PHM De Mulder[1], GON Oosterhof[2], C Bouffioux[3], AT van Oosterom[4], K Vermeylen[5] and R Sylvester[5] on behalf of the EORTC Genitourinary Group

[1]*Department of Medical Oncology, University Hospital Nijmegen, The Netherlands;* [2]*Department of Urology, University Hospital Nijmegen, The Netherlands;* [3]*Department of Urology, University of Liège, Liège, Belgium;* [4]*Department of Oncology, University Hospital Antwerp, Antwerp, Belgium;* [5]*EORTC Data Center, Brussels, Belgium.*

**Summary** In the treatment of renal cell carcinoma both complete (CRs) and partial remissions (PRs) have been obtained using recombinant (r) interferon alpha (IFN-$\alpha$), with response rates ranging from 0 to 31% (mean 16%). rIFN-$\gamma$ is a potent immunostimulating agent, but the clinical experience of its use is limited and results are conflicting. In a phase II study with the combination of rIFN-$\alpha_{2c}$ (Boehringer Ingelheim) and rIFN-$\gamma$ (Genentech, supplied by Boehringer Ingelheim) in 31 eligible patients, a response rate of 25% was recorded. Based on this observation a randomised phase III study was initiated to investigate the possible advantage of the addition rIFN-$\gamma$ to rIFN-$\alpha_{2c}$ treatment. Treatment consisted of rIFN-$\alpha_{2c}$ 30 $\mu$g m$^{-2}$ = 10 × 10$^6$ IU m$^{-2}$ s.c. twice weekly in arm A and the same dose of rIFN-$\alpha$ combined with rIFN-$\gamma$ 100 $\mu$g m$^{-2}$ = 2 × 10$^6$ IU m$^{-2}$ in arm B. Eligibility criteria included documented progression of disease; patients with bone lesions only and overt central nervous system metastases were excluded. Between November 1988 and September 1990, 102 patients were entered into the study. An interim analysis showed a response in 7/53 (13%) patients (two CRs and five PRs) in the rIFN-$\alpha_{2c}$ monotherapy arm and in 2/45 (4%) (one CR and one PR) patients in the combination arm. This difference was not statistically significant ($P = 0.17$). The probability of missing an eventual 10% advantage for the combination is 0.001. The numbers are insufficient to rule out a negative effect of the addition of rIFN-$\gamma$. The dose intensity of IFN-$\alpha_{2c}$ for the two treatment arms was the same. The addition of rIFN-$\gamma$ does not improve the response rate of rIFN-$\alpha_{2c}$ monotherapy. A possible detrimental effect cannot be excluded.

**Keywords:** renal cell carcinoma; interferon alpha; interferon gamma.

Patients with renal cell carcinoma (RCC) currently have few therapeutic options once the disease has become metastastic. Approximately 25% of such patients have metastatic disease at the time of first presentation (Ritchie *et al.*, 1983). The median survival for these patients is, independent of treatment, 6–12 months (De Forges *et al.*, 1988). Spontaneous regression of metastases after tumour nephrectomy occurs in less than 1% (Montie, 1977). Treatment with hormones and chemotherapy, both single agent and combination, has no proven impact on survival (Harris *et al.*, 1983; Yagoda and Bander, 1989). Several forms of immunotherapy have been applied, resulting in a limited number of sometimes durable responses (McCune, 1983). Interferon-alpha (IFN-$\alpha$) is most extensively used in the treatment of advanced RCC, both the natural and recombinant(r) forms. Most studies have provided evidence for modest but reproducible anti-tumour activity in advanced RCC (Goldstein and Laslo, 1986; Krown, 1987; Sarna *et al.*, 1987; Muss, 1988, Buzaid and Todo, 1989; Horoszewski and Murphy, 1989). The response rates recorded from adequate trials (i.e. more than 20 eligible patients and a dose of IFN-$\alpha$ of more than 3 × 10$^6$ U day$^{-1}$, $n = 431$) vary from 5 to 26% (mean 17%; 2% CR and 15% PR).

Experience with rIFN-$\gamma$ in renal cell carcinoma is limited and, with a few exceptions, disappointing (Rinehart *et al.*, 1986; Quesada *et al.*, 1987; Garnick *et al.*, 1988; Otto *et al.*, 1988; Aulitzky *et al.*, 1989; Bruntsch *et al.*, 1990). Little information is available about the optimal dose, schedule and route of IFN-$\gamma$ administration.

Modification of the host response is frequently restricted to a narrow dose range, and in a recent study optimal modulation by rIFN-$\gamma$ has been found in the low dose range (100 $\mu$g m$^{-2}$) (Maluish *et al.*, 1988). Against this background, the findings of Aulitzky *et al.* (1989) are interesting. They observed a 30% response rate (two CRs, four PRs) in 16 patients treated with 100 $\mu$g IFN-$\gamma$ (Genentech) s.c. once a week.

The combination of IFN-$\alpha$ and IFN-$\gamma$ has ben explored on the basis of *in vitro* observations indicating a synergism between rIFN-$\gamma$ and rIFN-$\alpha$ (Czarniecki *et al.*, 1984; Hubbell *et al.*, 1987). The results published so far are, however, disappointing (Kurzrock *et al.*, 1986; Foon *et al.*, 1988; Quesada *et al.*, 1988; Ernstoff *et al.*, 1990). De Mulder and co-workers (Geboers *et al.*, 1988; De Mulder *et al.*, 1990) studied the efficacy of the combination of an escalating dose rIFN-$\alpha_{2c}$ (6 $\mu$g m$^{-2}$ = 2 × 10$^6$ U m$^{-2}$ starting dose) and a fixed low dose of rIFN-$\gamma$ (100 $\mu$g m$^{-2}$ = 2 × 10$^6$ m$^{-2}$) twice weekly subcutaneously in patients with advanced progressive renal cell carcinoma. The overall response rate was 26% (two CRs, six PRs). The maximal tolerated dose of IFN-$\alpha_{2c}$ was 30 $\mu$g m$^{-2}$ (6–36 $\mu$g m$^{-2}$). The feasibility and efficacy of this approach was proven in the treatment of a second cohort of patients (De Mulder *et al.*, 1991). In view of these data, an EORTC randomised study was initiated to determine if the addition of rIFN-$\gamma$ has any impact on the response rate and survival of patients with advanced metastatic renal cell carcinoma.

### Patients and methods

### *Trial design*

The study was designed as a randomised phase III trial with an interim analysis planned after data for 40 eligible patients

Correspondence: PHM De Mulder, Department of Medical Oncology, University Hospital Nijmegen, PO Box 9101, 6500 HB Nijmegen, The Netherlands
Received 13 July 1994; revised 8 September 1994; accepted 23 September 1994

were available in each arm in order to ensure that continuation of the trial was ethical. After checking all eligibility criteria, the randomisation was centrally performed at the EORTC Data Center. Patients were stratified according to institution and performance status. The study was performed according to good clinical practice guidelines, which included the verification of all items given on the forms with the source documents. The main end points of the study were the comparison of the two treatments arms regarding response rate, time to response, response duration, survival and tolerance.

*Patient population*

Patients with histologically proven renal cell carcinoma with metastatic measurable or evaluable disease were considered for the study if they met the following criteria: age 18–75 years; no prior chemo- and or immunotherapy; prior hormonal treatment was allowed; there should have been proven progression, especially after a recent nephrectomy; World Health Organization (WHO) performance status 0–1; adequate haematological status, renal and liver function; normal serum calcium level; no concurrent serious medical illness (active infections, significant cardiac disease) or second malignancies except adequately treated basal cell carcinoma of the skin or cone biopsied carcinoma *in situ* of the cervix; no history of seizure disorders or signs of central nervous system metastases; life expectancy of at least 3 months; absence of a lipoprotein disorder. Concomitant medication with corticosteroids or vasodilators was not allowed. All patients gave their written or witnessed informed consent.

*Treatment regimen*

rIFN-$\alpha_{2c}$ and rIFN-$\gamma$ (Genentech) were supplied by Boehringer Ingelheim (Alkmaar, The Netherlands) and provided as a sterile lyophilised powder. The powder contained 15 $\mu$g of IFN-$\alpha_{2c}$ with a degree of purity of >98% and a specific activity of $4.4 \times 10^6$ IU 15 $\mu g^{-1}$ based on the NIH IFN-$\alpha$ standard G0-23-901-527 for rIFN-$\alpha$ or 150 $\mu$g of IFN-$\gamma$ with a specific activity of $2 \times 10^7$ IU $mg^{-1}$ protein, based on the NIH IFN-$\gamma$ standard Gg23-901-350. The freeze-dried preparations were reconstituted with 1 ml of sterile water immediately before use to yield rIFN-$\alpha_{2c}$ and rIFN-$\gamma$ concentrations of $5 \times 10^6$ IU $ml^{-1}$ and $10 \times 10^6$ IU $ml^{-1}$ respectively.

Injections were given subcuteneously twice a week on an out-patient basis, although it was recommended that the first injection be given during a brief stay in hospital. Treatment arm A consisted of rIFN-$\alpha_{2c}$ monotherapy and arm B consisted of the same dose of rIFN-$\alpha_{2c}$ plus rIFN-$\gamma$. rIFN-$\gamma$ was given at a dose of 100 $\mu g\, m^{-2}$ ($2 \times 10^6$ IU $m^{-2}$) and rIFN-$\alpha_{2c}$ was given at a dose of 30 $\mu g\, m^{-2}$ ($10 \times 10^6$ IU $m^{-2}$). In arm B the two IFNs were given at the same time at two different sites. In the case of WHO grade III–IV toxicity, treatment was discontinued until recovery, with a maximum delay in treatment of 14 days. In case of recovery, IFN treatment was to be restarted with a reduction of the dose of rIFN-$\alpha_{2c}$ of 6 $\mu g\, m^{-2}$. If a dose reduction resulted in a dose below 12 $\mu g\, m^{-2}$ the patient went off study. In case of grade II haematological toxicity, this was followed every 2 weeks until stabilisation was observed.

Acetaminophen (500 mg) was routinely prescribed to alleviate side-effects. This treatment was started 4 h before the IFN injection and continued for 24–48 h thereafter.

*Pretreatment and follow-up examinations*

Pre-study evaluations included full medical history and physical examination, tumour measurements, electrocardiogram, chest radiograph, white blood cell count, platelets and a complete chemistry profile. Four-weekly monitoring included side-effects according to the WHO grading system, haematological status, urine analyses and biochemical measures: creatinine, alkaline phosphatase, aspartate aminotransferase, alanine aminotransferase, lactate dehydrogenase, bilirubin, and gamma-glutamyl transferase. Total protein and subfractions, cholesterol, triglycerides and interferon antibodies were monitored every 4 weeks. Electrocardiography was repeated when indicated. Tumours were measured every 4 weeks with standard radiographic, computerised tomographic or ultrasonographic techniques as appropriate.

*Evaluation criteria*

The criteria for measurability of disease were according to the EORTC Data Center procedures manual (van Oosterom *et al.*, 1993). Nodes smaller than 2 cm and liver metastases smaller than 3 cm in diameter were not considered to be measurable or evaluable. Complete remission (CR) was defined as the disappearance of all clinical evidence of tumour for a minimum of 4 weeks. Partial remission (PR) was defined as a 50% or greater decrease in the sum of the products of the perpendicular diameters of all measurable lesions, without simultaneous increase in the size of any existing lesion or development of new lesions, for a minimum of 4 weeks. Progressive disease (PD) was defined as a 25% or greater increase in the size of at least one existing lesion or the appearance of new lesions. SD was defined as a decrease of less than 50% or an increase of less than 25%. For CR and PR duration of response was measured from the day of the start of treatment until disease progression or death. A patient was evaluable for toxicity when at least 4 weeks' treatment was given. A patient was evaluable for response when at least 8 weeks treatment was completed. However, all patients with PD, irrespective of the duration of treatment, were included in the response analysis. In case of stable disease treatment was to be discontinued after 6 months. When a PR or CR was seen treatment was to be continued to 1 year from the date of CR/PR.

*Statistical methods*

The expected response rate for the combination arm was 25–30%; the expected response rate for the monotherapy arm was between 15% and 20%. The minimal difference in response rate which was of practical interest was defined to be 15%. To detect such a difference at error rates $\alpha = 0.05$ and $\beta = 0.20$, 94 eligible and evaluable patients were required on each treatment. In order to ensure that continuation of the trial was ethical, an interum analysis was planned after receipt of the data for the first 40 patients in each arm. The response rates were compared using a two-sided Fisher exact test. The duration of response and the duration of survival were estimated by the Kaplan–Meier technique and compared using a two-sided log-rank test.

### Results

Between November 1988 and September 1990, 102 patients entered the study and were randomly assigned to treatment as follows: arm A, 54 patients; arm B, 48 patients. Four patients were ineligible, one on treatment A and three on treatment B: one patient had a second primary, one patient had no evaluable lesions and two patients started within 4 weeks after tumour nephrectomy without documented progression of metastatic disease. Nine eligible patients were not evaluable for response.

Patient characteristics at entry are depicted in Table I and are well balanced in the two treatment groups. For the entire group, 68% were male and 43% had a WHO performance status of 0. Eighteen per cent of the patients started treatment with the primary tumour *in situ*. Prior radiotherapy, mainly on threatening bone lesions, was given in 10% of the patients. Lung metastases were present in almost all patients. In 27% lung was the only site of disease. Liver metastases alone or in combination with other sites were seen in 14%. The majority of the patients had only one or two sites of disease (76%).

Table I Patient characteristics at entry

| | IFN-α | IFN-α + IFN-γ |
|---|---|---|
| No. of patients ($n$ = 102) | 54 | 48 |
| Not eligible | 1 | 3 |
| Male–female | 37:17 | 32:16 |
| Median age (range) | 55 (27–75) | 58 (32–74) |
| Performance status | | |
| WHO 0 | 20[a] | 23 |
| WHO 1 | 33 | 25 |
| Prior treatment ($n$ = 98) | | |
| Nephrectomy | 42 | 38 |
| No nephrectomy | 11 | 7 |
| Radiotherapy | 8 | 2 |
| Hormonal treatment | 0 | 1 |
| Site of disease | | |
| Lungs only | 14 | 14 |
| Lungs + primary | 4 | 3 |
| Lungs + nodes | 3 | 4 |
| Lungs + liver | 3 | 3 |
| Lungs + others | 7 | 9 |
| Liver + others | 5 | 3 |
| Number of sites | | |
| 1 | 17 | 19 |
| 2 | 19 | 23 |
| 3–6 | 16 | 9 |
| >5 | 0 | 1 |
| Not evaluable for response | 6 | 7 |

[a]Missing information for one patient.

Table II Response to treatment in eligible patients

| | rIFN-$\alpha_{2c}$ | rIFN-$\alpha_{2c}$ + IFN-γ |
|---|---|---|
| CR | 1 | 0 |
| pCR | 1 | 1 |
| PR | 5 | 1 |
| SD | 19 | 19 |
| PD | 22 | 20 |
| Early death | 2 | 3 |
| Unknown | 3 | 1 |
| Total | 53 | 45 |
| Response rate | 13% | 4% ($P$ = 0.17) |

There were four mixed responses on monotherapy and three mixed responses on the combination which are included in the Table as PD.

Figure 1 Time to progression.

Figure 2 Duration of survival.

*Treatment efficacy*

Considering all eligible patients (98) entered into the trial, the response rates were as follows: rIFN-$\alpha_{2c}$ monotherapy, one CR, one pathological CR, five PRs and 19 no change (NC), overall 13%; rIFN-$\alpha_{2c}$ + rIFN-γ, one pathological CR, one PR and 19 NC, overall 4% (Table II). This difference was not statistically significant at $P$ = 0.17 in favour of arm A. If a relevant difference in favour of the combination arm were 10% and the expected response on the monotherapy were 15%, the probability of missing this difference with the observed results would be 0.001. Although the difference was not statistically significant, the numbers are inadequate to show true equivalence or to exclude a potential negative effect of the addition of interferon-γ, however this was not the purpose of our study. A mixed response was seen in four out of 53 patients in arm A and three out of 45 in arm B. The median time to response among responders was 114 days (range 59–301 days) and the median response duration was 60 weeks, with seven of the nine responders having progressed. Based on an average follow-up of 1 year, the overall median survival was 43 weeks in arm A and 34 weeks in arm B ($P$ = 0.73) (Figure 1). The time to progression is given in Figure 2. When the patients with their primary *in situ* are excluded, the observed response rate was 7/42 (17%) for treatment arm A and 2/38 (5%) for arm B. The characteristics of all responding patients are shown in Table III. Six out of nine responded in the lungs, however only in two patients was this the only site of disease. In two patients concomitant metastases in the liver disappeared during therapy. The sites with unmeasurable disease remained clinically unchanged. Two patients had cytological proof of renal cell carcinoma in the enlarged node prior to the start of treatment. After discontinuation of treatment lymph node dissection was performed. Pathological examination revealed no tumour and the patients are therefore considered as having a pathological CR.

One possible explanation for the lack of response in the combination arm could be a difference in the dose intensity of rIFN-$\alpha_{2c}$ in the two groups. However, dose intensity, dose reductions and delays were similar in the two arms. In both arms 90% of the patients received 100% of the intended dose of rIFN-$\alpha_{2c}$.

*Toxicity*

Observed grade II and III toxicity is given in Table IV. Side-effects were those known to be associated with interferon treatment. The vast majority of the patients developed fever, anorexia, fatigue and to a lesser extent flu-like symptoms. There was no difference between the two treatment groups. One patient developed a WHO grade III thrombocytopenia, but fully recovered after discontinuation of treatment. The white blood count (WBC) was only marginally influenced, although in the combination arm three patients developed reversible WHO grade III leucopenia.

### Discussion

There is no doubt that interferons can induce responses in advanced renal cell carcinoma. The response percentage

Table III Characteristics of responding patients

| | Age | Sex | PS | Site | Size | Response | Comment | Response duration (weeks) |
|---|---|---|---|---|---|---|---|---|
| 1 | 61 | M | 1 | Lung | 22 × 19 | PR | PD in lungs and nodes, brain metastases. Dead due to malignant disease | 102 |
| | | | | Liver | 10 × 10 | CR | | |
| 2 | 41 | M | 0 | Lung | 23 × 22 | CR | PD supraclavicular node. CDF after lymph node dissection | 68 |
| | | | | Local | 29 × 29 | CR | | |
| 3 | 49 | M | 1 | Nodes | 30 × 35 | PR | PD lungs, brain metastases. Dead due to malignant disease | 45 |
| 4 | 46 | M | 0 | Nodes | 30 × 25 | pCR | No vital tumour at surgery | 192 + |
| 5 | 53 | F | 1 | Lung | 15 × 10 | PR | PD initial sites. Dead due to malignant disease | 21 |
| | | | | | 15 × 15 | | | |
| | | | | Bone | 40 × 20 | NC | | |
| 6 | 55 | M | 1 | Lung | 20 × 11 | CR | PD lungs. After metastasectomy NED. Brain metastases. After RT alive | 91 |
| | | | | | 13 × 16 | | | |
| | | | | | 8 × 9 | PR | | |
| | | | | Liver | 40 × 40 | | | |
| | | | | | 62 × 59 | NC | | |
| | | | | Bone | Irradiated | | | |
| 7 | 66 | F | 0 | Lung | 49 × 23 | PR | PD brain metastases. Death due to malignant disease | 52 |
| | | | | | 6 × 6 | | | |
| | | | | | 15 × 12 | | | |
| | | | | | 17 × 16 | | | |
| | | | | | 17 × 17 | | | |
| 8 | 65 | M | 0 | Lung | 23 × 20 | PR | PD initial sites. Dead due to malignant disease | 43 |
| | | | | | 15 × 13 | | | |
| | | | | | 18 × 15 | | | |
| | | | | | 20 × 18 | | | |
| 9 | 66 | F | 0 | Nodes | 25 × 40 | CR | After surgery only fibrosis and non-vital tumour was seen (necrosis) | 211 + |

CDF, continuously disease free; NED, no evidence of disease; RT, radiotherapy.

Table IV Toxicity (WHO grade)

| | $rIFN\text{-}\alpha_{2c}$ | | $rIFN\text{-}\alpha_{2c}$ + $IFN\text{-}\gamma$ | |
|---|---|---|---|---|
| | II | III | II | III |
| Platelets | 1 | 1 | 0 | 0 |
| WBC | 7 | 0 | 6 | 3 |
| Pulmonary | 4 | 2 | 0 | 1 |
| Fever | 27 | 9 | 24 | 9 |
| Cutaneous | 2 | 0 | 3 | 0 |
| Alopecia | 1 | 0 | 3 | 0 |
| Cardiac (rhythm) | 4 | 1 | 4 | 0 |
| Mucous | 1 | 0 | 2 | 0 |
| Nausea and vomiting | 11 | 5 | 9 | 5 |
| Neurotoxicity | 0 | 0 | 0 | 1 |
| Non-WHO | 2–3 | | 2–3 | |
| Flu-like syndrome | 16 | | 16 | |
| Anorexia | 21 | | 19 | |
| Mood alterations | 8 | | 6 | |
| Fatigue | 27 | | 24 | |
| Headache | 5 | | 8 | |

II, moderate, III, severe.

obtained from pooled data is about 17% (Krown, 1987; Muss, 1988; Horoszewicz *et al.*, 1989; De Mulder *et al.*, 1991). Prognostic factors such as performance status, tumour volume, presence of bone metastases and disease-free interval are well recognised and are the main explanation for the variation in response observed in the various studies. There is no indication that the route of administration, schedule or the type of IFN-α is critical for the observed clinical results. Dose dependency is suggested but an adequate randomised study to address this question has never been performed. Very low daily dosages, i.e. below $2 \times 10^6$ IU daily, are probably ineffective. Our own observation in a small group of patients corroborates this experience (Geboers *et al.*, 1988).

In the present multicentre study the activity of IFN-α is confirmed with an overall response rate of 13%. When only patients without their primary tumour are analysed, the response rate is 17%, which is consistent with the range observed in the literature. One should realise that these results were obtained with a relatively low dose of IFN-α ($10 \times 10^6$ IU $m^{-2}$) and a twice-weekly schedule, again an indication that the regimen is not critical and that IFN-α given above a certain threshold is able to induce responses in sensitive tumours. A remarkable finding was that in two patients an objective response in the liver was seen.

The main purpose of the study was to study the relevance of the addition of IFN-γ, which was based both on laboratory observations as well as on the results of earlier studies. The results were very disappointing because only in two patients (4%) was an objective response observed and the study, initially planned as a randomised phase III study, was stopped after an interim analysis. As indicated before, the probability of obtaining these results if a difference of 10% in favour of the combination was actually present is extremely low. Equivalence in outcome or even the inverse outcome, i.e. a potential adverse effect of the combination, cannot be excluded with adequate power in view of the numbers involved, but this was not the purpose of the study. There is no satisfactory explanation for this result. Patient characteristics of the two patient populations were similar and the likelihood that this observation could have been made by chance is almost negligible. The mechanisms of action of IFN-α are very pleiotropic, and many mechanisms can be responsible for the observed anti-tumour effect. There are actions directly on the tumour such as an antiproliferative effect, and there are indications that the induction of 2′,5′-oligoadenylate synthetase is related with this potential (Grander *et al.*, 1990). On the other hand, immunological properties such as the induction of natural killer activity and the enhancement of the expression of antigens on the tumour might play a role. The mechanism of action as elucidated in hairy cell leukaemia (Vedantham *et al.*, 1992), the carcinoid (Grander *et al.*, 1990) and the observation that the addition of 20 mg of prednisone had no impact on the anti-tumour effect (Fossa *et al.*, 1990) suggest a direct effect on the tumour cell. Interferon-gamma is considered a true immunomodulating agent, predominantly on macrophages, with few direct antiproliferative effects on tumour cells. The results with IFN-γ

monotherapy are generally disappointing. The 30% response rate observed by Aulitzky *et al.* (1989), so far unconfirmed, applying an individually tailored dose of IFN-γ based on parameters of immune stimulation such as neopterin excretion, indicates the sensitivity of this disease depending on very specific requirements. The IFN-γ dose used in the present study was within the same range. One of the explanations of the generally low response rate in combination studies could be the relatively low dose of IFN-α given in these studies (De Mulder *et al.*, 1991). In the present study this explanation is unlikely in view of the almost identical dose intensity of IFN-$\alpha_{2c}$ in the two treatment arms.

Based on these results, the combination of IFN-α and IFN-γ in the dose and schedule described in this study cannot be recommended. Our results confirm the limited activity of IFN-α monotherapy in this disease.

**References**

AULITZKY W, GASTL WE, AULITZKY WE, HEROLD M, KEMMLER J, MULL B, FRICK J AND HUBER C. (1989). Successful treatment of metastatic renal cell carcinoma with a biological active dose of recombinant interferon-gamma. *J. Clin. Oncol.*, **7**, 1875-1884.

BRUNTSCH U, DE MULDER PHM, TEN BOKKEL HUININK WW, CLAVEL M, DROZD K, KAY SB, RENARD J AND VAN GLABBEKE M. (1990). Phase II study of recombinant human interferon-gamma in metastatic renal cell carcinoma. *J. Biol. Response Mod.*, **9**, 335-338.

BUZAID AC AND TODD MB. (1989). Therapeutic options in renal cell carcinoma. *Semin. Oncol.*, **16**, 12-19.

CZARNIECKI CW, FENNIE CW, POWERS DB AND ESTELL DA. (1984). Synergistic antiviral and anti proliferative activities of *E. coli* derived human alpha, beta, and gamma interferon. *J. Virol.*, **49**, 490-496.

DE FORGES A, REY A, KLINK M, GHOSN M, KRAMAR A AND DROZ JP. (1988). Prognostic factors in adult metastatic renal carcinoma: a multivariate analysis. *Semin. Surg. Oncol.*, **4**, 149-154.

DE MULDER PHM, DEBRUYNE FMJ, FRANSSEN MPH, GEBROERS AD, STRIJK S, REINTJES AG, DOESBURG WH AND DAMSMA O. (1990). Phase I/II study of recombinant interferon alpha and gamma in advanced progressive renal cell carcinoma. *Cancer Immunol. Immunother.*, **31**, 321-324.

DE MULDER PHM, FRANSSEN MPH, PUNT CJA AND DEBRUYNE FMJ. (1991). Monotherapy and combination therapy with interferon-alpha, interferon-gamma and tumor necrosis factor-alpha in metastatic renal cell carcinoma. In *Immunotherapy of Renal Cell Carcinoma, Clinical and Experimental Developments*, Debruyne FMJ, Bukowski RM, Pontes JE and De Mulder PHM (eds) pp. 82-90. Springer: Berlin.

ERNSTOFF MS, NAIR S, BAHNSON RR, MIKETIC LM, BANNER B, GOODING W, CAY R, WHITESIDE T, HAKALA T AND KIRKWOOD JM. (1990). A phase Ia trial of sequential administration recombinant DNA-produced interferons: combination recombinant interferon-gamma and recombinant interferon-alfa in patients with metastatic renal cell carcinoma. *J. Clin. Oncol.*, **8**, 1637-1649.

FOSSA SD, GUNDERSON R AND MOE B. (1990). Recombinant interferon-alpha combined with prednisone in metastatic renal cell carcinoma. Reduced toxicity without reduction of the response rate - a phase II study. *Cancer*, **65**, 2451-2454.

FOON K, DOROSHOW J, BONNEM E, FEFER A, GRAHAM S, GROSH B, NARAYAN P, ELIAS L, HARVEY H AND SCHULOF R. (1988). A prospective randomized trial of α2b-interferon γ-interferon or the combination in advanced metastatic renal cell carcinoma. *J. Biol. Response Mod.*, **7**, 540-545.

GARNICK MB, REICH SD, MAXWELL B, COVAL-GOLDSMITH S, RITCHIE JP AND RUDNICK SA. (1988). Phase I/II study of recombinant interferon gamma in advanced renal cell carcinoma. *J. Urol.*, **139**, 251-255.

GEBOERS ADH, DE MULDER PHM, DEBRUYNE FMJ, STRIJK SP AND DAMSMA O. (1988). Alpha and gamma interferon in the treatment of advanced renal cell carcinoma. *Semin. Surg. Oncol.*, **4**, 191-194.

GOLDSTEIN D AND LASLO J. (1986). Interferon therapy in cancer: from imaginon to interferon. *Cancer Res.*, **46**, 4315-4329.

GRANDER D, OBERG K, LUNDQVIST M, JANSON ET, ERIKSSON B AND EINHORN S. (1990). Interferon induced enhancement of 2',5'-oligoadenylate synthetase in mid-gut carcinoid tumours. *Lancet*, **36**, 337-340.

HARRIS DT. (1983). Hormonal therapy and chemotherapy of renal cell carcinoma. *Semin. Oncol.*, **10**, 422-430.

HOROSZEWICZ S AND MURPHY GP. (1989). An assessment of the current use of human interferons in therapy of urological cancers. *J. Urol.*, **142**, 1173-1180.

HUBBELL HR, CRAFT JA, LEIBOWITZ PH AND GILLESPIE DH. (1987). Synergistic antiproliferative effect of recombinant alpha-interferons with recombinant gamma-interferons. *J. Biol. Response Mod.*, **6**, 141-153.

KROWN SE. (1987). Interferon treatment of renal cell carcinoma. Current status and future prospects. *Cancer*, **59**, 647-651.

KURZROCK R, ROSENBLUM MG, QUESADA JR, SHERWIN SA, ITRI LM AND GUTTERMAN JU. (1986). Phase I study of a recombinant interferon-alph and recombinant-gamma in cancer patients. *J. Clin. Oncol.*, **4**, 1677-1683.

MALUISH, AE, URBA WJ, LONGO DL, OVERTON WR, COGGIN D, CRISP ER, WILLIAMS R, SHERWIN SA, GORDON J AND STEIS RG. (1988). The determination of an immunologically active dose of interferon gamma in patients with melanoma. *J. Clin. Oncol.*, **6**, 434-445.

MCCUNE CS. (1983). Immunologic therapies in kidney carcinoma. *Semin. Oncol.*, **10**, 431-436.

MONTIE JE, STEWART BH, STRAFFON RA, BANOWSKY LH, HEWITT CB AND MONTAGUE DK. (1977). The role of adjunctive nephrectomy in patients with metastatic renal cell carcinoma. *J. Urol.*, **117**, 272-275.

MUSS HB. (1988). Interferon therapy of metastatic renal cell cancer. *Semin. Surg. Oncol.*, **4**, 199-203.

OTTO U, CONRAD S, SCHNEIDER AW AND KLOSTERHALFEN H. (1988). Recombinant interferon gamma in the treatment of metastatic renal cell carcinoma. *Arzneim. Forsch. Drug Res.*, **38**, 38, 1658.

QUESADA JR, KURZROCK R, SHERWIN SA AND GUTTERMAN JU. (1987). Phase II studies of recombinant human interferon gamma in metastatic renal cell carcinoma. *J. Biol. Response Mod.*, **6**, 20-27.

QUESADA JR, EVANS L, SAKS SR AND GUTTERMAN JU. (1988). Recombinant interferon alpha and gamma in combination as treatment for metastatic renal cell carcinoma. *J. Biol. Response Mod.*, **7**, 234-239.

RINEHART JJ, MALSPEIS L, YOUNG D AND NEIDHART JA. (1986). Phase I/II trial of human recombinant interferon gamma in renal cell carcinoma. *J. Biol. Response Mod.*, **5**, 300-308.

RITCHIE AWS AND CHRISHOLM GD. (1983). The natural history of renal cell carcinoma. *Semin. Oncol.*, **10**, 390-400.

SARNA G, FIGLIN R AND DEKERNION J. (1987). Interferon in renal cell carcinoma. The UCLA experience. *Cancer*, **59**, 610-612.

VAN OOSTEROM AT, TANNOCK I, MATSUMURA Y, AKAZA H, EINSTEIN M, HAFERMANN M, HALL R, HIRAO Y, JONES W, KOONTZ W, MURPHY G, RAGHAVAN D, OGAWA M, SPLINTER T, STOTER G AND WAJSMAN Z. (1993). Response criteria in phase II/phase III studies of invasive bladder cancer. In *Consensus Development in Clinical Bladder Cancer Research*, Proceedings of the Second and Third International Consensus Development Symposia, Niijima T, Aso Y, Koontz W, Prout G and Denis L. (eds) pp. 17-26 Brussels.

VEDANTHAM S, GAMLIEL H AND GOLOMB HM. (1992). Mechanism of interferon action in hairy cell leukemia: a model of effective cancer biotherapy. *Cancer Res.*, **52**, 1056-1066.

YAGODA A AND BANDER NH. (1989). Failure of cytotoxic chemotherapy, 1983-1988, and the emerging role of monoclonal antibodies for renal cancer. *Urol. Int.*, **44**, 338-345.